# Venous Thromboembolism Prophylaxis Compliance in Orthopedic Patients With Cardiac Risk Factors

**DOI:** 10.7759/cureus.79772

**Published:** 2025-02-27

**Authors:** Usama Asif, Usman Ghani, Sheikh Muhammad Rehman Zia, Farhan Majeed, Muhammad Usman, Aqsa Amjad, Humna Khan, Bilal Qammar

**Affiliations:** 1 Internal Medicine, Aziz Bhatti Shaheed Teaching Hospital, Gujrat, PAK; 2 Cardiology, Army Cardiac Hospital, Lahore, PAK; 3 Orthopaedics, Lahore General Hospital, Lahore, PAK; 4 Internal Medicine, Jinnah Hospital, Lahore, PAK; 5 Obstetrics and Gynaecology, Shalamar Hospital, Lahore, PAK; 6 Internal Medicine, Fatima Memorial Hospital, Lahore, PAK; 7 Internal Medicine, Shalamar Hospital, Lahore, PAK

**Keywords:** compliance, deep vein thrombosis, prophylaxis, pulmonary embolism, venous thromboembolism

## Abstract

Introduction

Venous thromboembolism (VTE) is a critical condition that includes deep vein thrombosis (DVT) and pulmonary embolism (PE), both of which can lead to severe complications, including death, if not adequately managed.

Objective

This study aimed to evaluate adherence to prophylactic measures and identify factors influencing compliance.

Methodology

This study was done in Shalamar Hospital, Lahore, from October 2023 to October 2024. Baseline assessments were conducted to gather demographic and clinical data, including age, gender, body mass index (BMI), comorbidities, and cardiac risk factors such as atrial fibrillation or coronary artery disease. Details of the surgical procedures, including type, duration, and anesthesia used, were also recorded. Compliance with VTE prophylaxis was monitored through patient interviews, direct observation, and medical record reviews. Pharmacological prophylaxis compliance involved evaluating the administration of anticoagulants regarding dosage, timing, and frequency. Mechanical prophylaxis adherence was assessed by monitoring the use of compression stockings and pneumatic devices.

Compliance was measured as the percentage of patients who adhered to the prescribed prophylaxis protocols. Primary outcomes included the incidence of VTE events, such as DVT or PE, within 30 days post-surgery. Follow-ups were conducted through in-person visits or telephonic interactions to monitor compliance and postoperative outcomes.

Data were analyzed using IBM SPSS Statistics for Windows, Version 26.0 (Released 2019; IBM Corp., Armonk, New York, United States). Continuous variables, such as age and BMI, were summarized using means and standard deviations, while categorical variables, like compliance rates, were expressed as frequencies and percentages. Subgroup analyses were conducted to identify variations in compliance rates based on demographic and clinical factors using t-tests.

Results

The study included 90 patients, with a mean age of 58.9±8.4 years and a nearly equal gender distribution (54% male and 46% female). The mean BMI of the participants was 28.5±3.2 kg/m². Hypertension was the most common cardiac risk factor, affecting 67% (n=60) of patients, followed by atrial fibrillation (22%; n=20) and heart failure (11%; n=10). Among the surgeries, 65% (n=58) were elective, while 35% (n=32) were emergency procedures. VTE incidence was 6.7% (n=6), with 4.4% (n=4) developing DVT and 2.2% (n=2) experiencing PE. Bleeding complications occurred in 8.9% (n=8) of patients, with 6.7% (n=6) experiencing minor bleeding and 2.2% (n=2) reporting major bleeding. Patients undergoing elective surgeries exhibited higher pharmacological compliance at 78% (n=45) and mechanical compliance at 88% (n=51), resulting in an overall compliance rate of 84% (n=49). In contrast, patients undergoing emergency surgeries had lower compliance rates, with 63% (n=20) for pharmacological prophylaxis and 69% (n=22) for mechanical prophylaxis, leading to an overall compliance rate of 63% (n=20).

Conclusion

It is concluded that compliance with VTE prophylaxis plays a pivotal role in reducing the risk of thromboembolic events in orthopedic patients with cardiac risk factors.

## Introduction

Venous thromboembolism (VTE) is a critical condition that includes deep vein thrombosis (DVT) and pulmonary embolism (PE), both of which can lead to severe complications, including death, if not adequately managed. Patients undergoing major orthopedic surgery, including elective procedures such as knee or hip replacement, repair, or fracture treatment, are considered at high risk for VTE [1]. This added risk results from staying inactive for long periods, damage to the tissues, and inflammation commonly related to operations. If there are added cardiac risk factors like atrial fibrillation, hypertension, heart failure, or coronary artery disease, then the risk multiplies significantly [2]. Underlying risk factors associated with hypertension intensify the hypercoagulable state by attributing to reduced blood flow and endothelial dysfunction to form a multi-mediated structural base of clot formation. As such, these patients need thorough preventive goals to decrease the occurrence of VTE [3]. Therefore, the foundation of VTE prevention can be successfully based on the wide utilization of pharmacological and mechanical anticoagulant approaches. Pharmacological management focuses on anticoagulation therapy including low-molecular-weight heparin (LMWH), direct oral anticoagulants (DOACs), or vitamin K antagonists to prevent clots from forming and minimize the risk of thromboembolism. Graduated compression stockings (GCS) and intermittent pneumatic compression devices (IPCDs) are mechanical techniques by which a patient's venous return and stasis can be improved to a greater extent as compared to pharmacotherapy [4].

Adhering to prescribed guidelines for VTE prophylaxis is essential for achieving optimal outcomes. However, compliance is still an issue of concern due to several reasons. Patients with cardiac comorbidities have an increased risk for bleeding complications and therefore may deny anticoagulant therapies or even become discouraged from following their use [5]. Furthermore, the challenge exists in achieving the balance between thrombosis prevention and bleeding risk in treatment, especially if the patient is elderly and has other chronic diseases. Other perceived challenges affecting the level of compliance include physical constraints like the availability of compression devices or the inability of the patient to acquire enough knowledge about the best techniques in medication usage [6]. The consequences of non-compliance for clinical practice are staggering. VTE with no strict compliance to the use of prophylaxis results in either rapidly fatal PE or long-term complications such as post-thrombotic syndrome (PTS). Furthermore, VTE complicates the clinical condition of the patient and increases medical expenses because of associated longer hospitalization, hospital readmissions, and critical care requirements. Hence, enhancement of VTE prophylaxis concussion is a clinical and economically relevant goal [7].

To achieve good adherence levels, therefore, a systems approach needs to be employed. This includes proper stratification for the risk of patients to ensure they receive the preventive measures in the right manner and reduce the complications arising as much as possible. For example, those patients at high risk of bleeding may require mechanical prophylaxis, either as monotherapy or in combination with lesser anticoagulation intensities [8]. Certain measures such as the audit of prophylaxis protocols, staff education, and compliance with the available proof-based guidelines must be carried out periodically. Moreover, the general care plan that considers the distinctive features with regard to part risks and preferences would also enhance the results while decreasing the effects of complications. VTE prophylaxis management is an unwiring principle in caring for orthopedic patients especially those with risk factors for cardiovascular diseases [9]. Given the multifactorial nature of surgical, immobility, and cardiac risks, their prevention requires a blended and patient-specific intervention strategy. In this context, when offering solutions to the challenges towards adherence, perfect education, collaboration of a multidisciplinary team, and advanced application of technology would reduce the occurrence rate of VTE, enhance the quality of the patient's health, and decrease the overall costs of the healthcare system.

This study aims to evaluate adherence to prophylactic measures and identify factors influencing compliance.

## Materials and methods

This study was conducted at Shalamar Hospital located in Lahore, Pakistan, from October 2023 to October 2024 after obtaining approval from the Institutional Review Board of Shalamar Medical and Dental College (approval number: SMDC-IRB/AL/2023-092). This study contains the data of 90 patients.

Eligible participants were orthopedic patients with complete medical records and ≥18 years of age with documented cardiac risk factors, including hypertension, atrial fibrillation, and heart failure. Patients were included regardless of whether they underwent elective or emergency orthopedic procedures, as long as they were at risk for VTE and receiving prophylactic treatment.

Exclusion criteria encompassed individuals who had contraindications to pharmacological or mechanical VTE prophylaxis, such as active bleeding disorders, severe thrombocytopenia, or a known hypersensitivity to anticoagulants. Additionally, patients with missing or insufficient medical records, those with a history of prior VTE, those already on long-term anticoagulation therapy before surgery, and individuals with severe renal or hepatic impairment that could affect anticoagulant metabolism were excluded. The study also did not include pregnant patients and individuals with malignancies requiring ongoing chemotherapy.

Baseline assessments were conducted to gather demographic and clinical data, including age, gender, body mass index (BMI), comorbidities, and cardiac risk factors such as atrial fibrillation or coronary artery disease. Details of the surgical procedures, including type, duration, and anesthesia used, were also recorded. Compliance with VTE prophylaxis was monitored through patient interviews, direct observation, and medical record reviews. Pharmacological prophylaxis compliance involved evaluating the administration of anticoagulants regarding dosage, timing, and frequency. Mechanical prophylaxis adherence was assessed by monitoring the use of compression stockings and pneumatic devices.

Compliance was measured as the percentage of patients who adhered to the prescribed prophylaxis protocols. Primary outcomes included the incidence of VTE events, such as DVT or PE, within 30 days post-surgery. Follow-ups were conducted through in-person visits or telephonic consultations to monitor compliance and postoperative outcomes.

Data were analyzed using IBM SPSS Statistics for Windows, Version 26.0 (Released 2019; IBM Corp., Armonk, New York, United States). Continuous variables, such as age and BMI, were summarized using means and standard deviations, while categorical variables, like compliance rates, were expressed as frequencies and percentages. Subgroup analyses were conducted to identify variations in compliance rates based on demographic and clinical factors using t-tests.

## Results

The study included 90 patients, with a mean age of 58.9±8.4 years and a nearly equal gender distribution (54% male and 46% female). The mean BMI of the participants was 28.5±3.2 kg/m². Hypertension was the most common cardiac risk factor, affecting 67% (n=60) of patients, followed by atrial fibrillation (22%; n=20) and heart failure (11%; n=10). Among the surgeries, 65% (n=58) were elective, while 35% (n=32) were emergency procedures. VTE incidence was 6.7% (n=6), with 4.4% (n=4) developing DVT and 2.2% (n=2) experiencing PE. Bleeding complications occurred in 8.9% (n=8) of patients, with 6.7% (n=6) experiencing minor bleeding and 2.2% (n=2) reporting major bleeding (Table 1).

**Table 1:** Baseline characteristics of the study population VTE: venous thromboembolism; BMI: body mass index

Characteristic	Value
Total patients	90
Mean age (years)	58.9±8.4
Gender (male/female)	49/41
Mean BMI (kg/m²)	28.5±3.2
Cardiac risk factors	
- Hypertension	67% (n=60)
- Atrial fibrillation	22% (n=20)
- Heart failure	11% (n=10)
Surgery type	
- Elective	65% (n=58)
- Emergency	35% (n=32)
Total VTE cases	6.7% (n=6)
- Deep vein thrombosis	4.4% (n=4)
- Pulmonary embolism	2.2% (n=2)
Bleeding complications	8.9% (n=8)
- Minor bleeding	6.7% (n=6)
- Major bleeding	2.2% (n=2)

Pharmacological prophylaxis showed a compliance rate of 72% (n=65), while mechanical prophylaxis had a higher compliance rate of 81% (n=73). Patients receiving combined pharmacological and mechanical prophylaxis demonstrated the highest compliance at 85% (n=38). In contrast, those receiving only a single type of prophylaxis (either pharmacological or mechanical) exhibited a lower compliance rate of 67% (n=31), highlighting the effectiveness of a multimodal approach in improving adherence (Table 2).

**Table 2:** Compliance with VTE prophylaxis VTE: venous thromboembolism

Prophylaxis type	Compliance rate	Number of patients
Overall compliance	77%	69
Pharmacological prophylaxis	72%	65
Mechanical prophylaxis	81%	73
Combined pharmacological and mechanical prophylaxis	85%	38
Single prophylaxis (either one)	67%	31

Table 3 shows that patients undergoing elective surgeries had a higher compliance rate of 84% (n=49) compared to 63% (n=20) for emergency surgeries (p=0.03). Age also influenced adherence, with patients under 65 years showing a compliance rate of 81% (n=43) compared to 72% (n=26) for those aged 65 years or older (p=0.05). Similarly, patients with fewer comorbidities demonstrated better compliance at 82% (n=56), whereas those with multiple comorbidities had a compliance rate of 67% (n=13) (p=0.04).

**Table 3:** Factors influencing compliance Independent t-test (for comparing compliance rates across patient factors)

Factor	Compliance rate	Test statistic (t-value)	P-value
Elective surgery	84% (n=49)	2.27	0.03
Emergency surgery	63% (n=20)	-	-
Age <65 years	81% (n=43)	2.00	0.05
Age ≥65 years	72% (n=26)	-	-
Fewer comorbidities	82% (n=56)	2.15	0.04
Multiple comorbidities	67% (n=13)	-	-

Table 4 shows that patients undergoing elective surgeries exhibited higher pharmacological compliance at 78% (n=45) and mechanical compliance at 88% (n=51), resulting in an overall compliance rate of 84% (n=49). In contrast, patients undergoing emergency surgeries had lower compliance rates, with 63% (n=20) for pharmacological prophylaxis and 69% (n=22) for mechanical prophylaxis, leading to an overall compliance rate of 63% (n=20). When combining both groups, pharmacological compliance was 72% (n=65), mechanical compliance was 81% (n=73), and the overall compliance rate was 77% (n=69).

**Table 4:** VTE prophylaxis compliance by surgical type VTE: venous thromboembolism

Surgical type	Pharmacological compliance	Mechanical compliance	Overall compliance
Elective surgery	78% (n=45)	88% (n=51)	84% (n=49)
Emergency surgery	63% (n=20)	69% (n=22)	63% (n=20)
Combined (both groups)	72% (n=65)	81% (n=73)	77% (n=69)

Table 5 shows that emergency surgery was associated with a significantly higher likelihood of non-compliance, with an odds ratio (OR) of 2.1 (95% CI: 1.1-4.2; p=0.02). Patients with multiple comorbidities also had an increased risk of non-compliance (OR: 1.8; 95% CI: 1.0-3.3; p=0.04). Inadequate patient education emerged as the strongest predictor, with an OR of 3.2 (95% CI: 1.5-6.7; p=0.01). Although older age (≥65 years) was associated with a higher risk (OR: 1.5; 95% CI: 0.9-2.6), it did not reach statistical significance (p=0.07).

**Table 5:** Predictors of non-compliance

Predictor	Odds ratio	95% confidence interval	P-value
Emergency surgery	2.1	1.1-4.2	0.02
Multiple comorbidities	1.8	1.0-3.3	0.04
Inadequate patient education	3.2	1.5-6.7	0.01
Age ≥65 years	1.5	0.9-2.6	0.07

## Discussion

The findings of this study highlight the importance of adherence to VTE prophylaxis in orthopedic patients with cardiac risk factors, emphasizing the need for targeted interventions to improve compliance. The level of compliance with VTE prophylaxis at our facility was 77% with significantly higher compliance in elective surgeries than in emergency surgeries. More than 60% of the patients had a high risk of VTE, and this highlights the difficulties of implementing early prevention in postoperative patients, especially those in urgent surgical operations where logistic and time factors are likely to influence compliance with the recommended preventive measures [9]. Non-adherence to the VTE prophylaxis guidelines was again confirmed by the investigations to be a significant predictor of poor outcomes [10]. Of the confirmed VTE, two patients were of male gender, and six of the six were VTE non-compliant; therefore, there is an absolute association of non-compliance with VTE thromboembolic event occurrence. This is in concordance with similar investigations arguing that, if preventive measures are well put into practice, VTE risk in high-risk persons can be greatly reduced [11]. Social determinants of health (SDOH) significantly impact VTE risk, prevention, and management. Factors such as socioeconomic status, healthcare access, education, and racial or geographic disparities contribute to differences in incidence and outcomes. Patients from lower-income backgrounds often face barriers to timely diagnosis and treatment due to financial constraints, limited healthcare access, and lack of awareness. Disparities in healthcare resources and systemic biases also affect prophylaxis adherence, increasing VTE-related complications in underserved populations. Environmental and occupational factors, like sedentary lifestyles and poor living conditions, further elevate risk [12]. Addressing these disparities through public health initiatives, improved access to anticoagulation therapy, and patient education can help reduce VTE-related morbidity and mortality. An awareness level of the patients was again found to be another significant compliance factor. The authors established that patient's knowledge of the vital aspects in terms of taking preventive measures was reasonably fair, especially in those patients, who were found to have good compliance with the appointed containing low levels of exigency measures [13]. This calls for the integration of proper manners of teaching the patients through counseling, drawing figures and arrows, and following up with the patients through constant reminders. Mobile applications and automated alerts in particular are equally aligned with improving the rate of adherence [14]. This type of non-compliance was also found to have specific predictors like those that involved emergent surgical procedures, with multiple comorbidities and perceived poor understanding among patients concerning medications. Such results imply that different preventive approaches are needed to reach out to these high-risk populations [15]. For example, those who are admitted with clear surgical emergency indications can be facilitated by standardized protocols for VTE prevention at the time of surgical planning. Likewise, communication between surgeons, cardiologists' nurses, and other members of the healthcare team could assist in managing patients with polymorbidity [16]. Bleeding complications overall were noted in 8.9% of patients, while severe bleeding was noted in 2.2%. This underlines the need to weigh the potential adverse effects and benefits of pharmacological prevention, especially, in patients with existing cardiovascular disease risks. Currently, there are several tools for risk stratification like the Caprini Risk Assessment Model that can allow choosing a more individual approach to the use of preventive measures [17]. Higher compliance rates regarding the mechanical prophylaxis (81%) in contrast to the pharmacological one (72%) indicate that patients do not consider the mechanical approach as dangerous as the pharmacological one [18]. The co-integration of the two showed the best compliance (85%) and best outcomes in the prevention of VTE. Such an outcome confirms the existing trends of the multimodal approach to VTE prevention. The study has the following implications in clinical practice. First, it emphasizes compliance with VTE and the need for healthcare organizations to use VTE prophylaxis guidelines in high-risk populations. Second, it communicates the importance of going back to the drawing board and correcting patient-centered approaches that might be derailed by reasons such as fear of side effects, lack of understanding, or practical challenges.

This study provides valuable insights into compliance with VTE prophylaxis; however, it has several limitations. First, this was a single-centered study, which may limit the generalizability of the findings to other settings. Second, the sample size (90 patients), while sufficient for preliminary analysis, may not be large enough to detect smaller but clinically significant differences in compliance rates. Third, self-reported compliance and observational monitoring may introduce reporting bias, as patients might overestimate adherence and healthcare providers may have inconsistencies in documentation. Fourth, confounding variables, such as differences in postoperative care, staff training, and institutional protocols, were not fully controlled, which may have influenced compliance outcomes. Finally, follow-up was conducted primarily through in-person visits and phone calls, which might have led to the underreporting of VTE events due to patient recall bias or loss to follow-up. Future studies with larger, multicenter cohorts, objective compliance measures, and longer follow-up durations are needed to validate these findings and improve the understanding of compliance determinants in VTE prophylaxis.

## Conclusions

Adherence to VTE prophylaxis is essential in reducing thromboembolic risk in orthopedic patients with cardiac comorbidities. The study found that compliance was higher in elective surgeries, likely due to better preoperative planning and structured protocols, while emergency procedures faced challenges due to time constraints and acute medical conditions. Patients with multiple comorbidities also exhibited lower adherence, highlighting the need for individualized risk assessments and multidisciplinary collaboration to ensure optimal preventive care.

Patient education emerged as a key factor in compliance, with those more informed about VTE prophylaxis demonstrating better adherence. Structured educational initiatives, digital health tools, automated reminders, and counseling programs can enhance patient engagement and improve compliance rates. Strengthening perioperative management and implementing standardized VTE prevention protocols, particularly for emergency surgical patients, is crucial in reducing the burden of thromboembolic events.
